# Nurses’ ethical decision-making during end of life care in South Korea: a cross-sectional descriptive survey

**DOI:** 10.1186/s12910-021-00665-9

**Published:** 2021-07-16

**Authors:** Arum Lim, Sanghee Kim

**Affiliations:** 1 Department of Nursing, Graduate School, Yonsei University, Seoul, Korea; 2 College of Nursing and, Mo-Im Kim Nursing Research Institute, Yonsei University, 50-1 Yonsei-ro, Seodaemun-gu, Seoul, 03722 South Korea

**Keywords:** Nurse, Ethics, Decision-making, End-of-life, Moral sensitivity, Ethical decision

## Abstract

**Background:**

Although nurses are crucial to ensure patients’ peaceful death in hospitals, many nurses experience various ethical conflicts during end-of-life care. Therefore, research on nurses’ entire ethical decision-making process is required to improve nurses’ ethical decision-making in end-of-life care. This study aimed to identify Korean nurses’ ethical decision-making process based on their moral sensitivity to end-of-life patients.

**Methods:**

In total, 171 nurses caring for terminal patients responded to the survey questionnaire. To measure the participants’ moral sensitivity and ethical decision-making process, we used the Korean version of the Moral Sensitivity Questionnaire and Nurses’ Ethical Decision-Making around End of Life Care Scale. Finally, multiple linear regression analysis was used to investigate the effect of moral sensitivity on nurses’ ethical decision-making.

**Results:**

The mean of moral sensitivity was 4.8 ± 0.5 (out of 7), and that of ethical decision-making was 4.6 ± 0.5 (out of 6). Among the sub-dimensions of ethical decision-making, the highest score was in perceived professional accountability (5.2 ± 0.5), and the lowest in moral reasoning and moral agency (3.9 ± 0.6); the score of moral practice was 4.4 ± 0.7. In the multiple linear regression model, moral sensitivity (β = 0.852, *p* < .001), clinical department (β =  − 7.018, *p* = .035), ethics education (β = 20.450, *p* < .001), job satisfaction (β = 5.273, *p* < .001), and ethical conflict (β =  − 2.260, *p* = 0.031) were influential ethical decision-making factors.

**Conclusions:**

This study revealed a gap between nurses’ thoughts and practices through the ethical decision-making process. They failed to lead their thought to moral practice. It also implies that moral sensitivity could positively affect nurses’ ethical decision-making. To make nurses morally sensitive, exposing them to various clinical cases would be helpful. Additionally, ethics education and clinical ethics supporting services are valuable for improving nurses’ ethical decision-making. If nurses improved their ethical decision-making regarding end-of-life care, their patients could experience a better quality of death.

## Background

As medical technology advancements have led to improvements in life-sustaining treatment (LST), the number of people who die in hospitals has increased in South Korea. In 2018, more than three-fourths (76.2%) of the total number of deaths occurred in a medical setting [1]. Nevertheless, patients still tend to be excluded from their end-of-life (EOL) decision-making. Most of the decisions requiring a do-not-resuscitate order are made by the patients’ healthcare providers or family [2, 3], and many patients spend their time undergoing various LSTs even if they are unlikely to recover [4]. However, the number of people who want to die peacefully is considerable [5–7]. In Korea, after the Act on Hospice and Palliative Care and Decisions on LST for EOL Patients was enforced in 2018 [8], the number of registrants for advance directives has gradually increased [9].

Nurses play an essential role in improving patients’ experience nearing end of life. According to the middle range theory, a peaceful end of life means patients do not experience pain but comfort and dignity [10]. Nurses satisfy patients’ needs for optimal physical care, such as pain management, and promote a peaceful environment [11]. Further, they provide emotional and spiritual support to their patients and families [11]. When patients and their families consider withdrawing LST, nurses collaborate with multidisciplinary teams to ensure that patients have accurate information regarding their decisions [12]. Thus, they encourage their patients to undergo peaceful death with dignity [13].

However, in many cases, determining the best course of action regarding a patient’s EOL care is challenging [14], as such decisions usually directly affect a patient’s life and death [15]. As a result, nurses who take care of EOL patients sometimes face ethical dilemmas, such as the futility of treatments, physician conflicts, confronting dying patients, or staffing shortages causing low-quality EOL care [14]. In cases where nurses cannot properly deal with ethical dilemmas or conflicts, they may experience extreme stress [16] and decreased quality of care [17]. Therefore, the ethical decision-making process of professional nurses should be investigated in-depth.

Nurses’ ethical decision-making is defined as a sequential process consisting of professional accountability and moral components, such as moral sensitivity, judgment, motivation, and behavior [18]. Professional accountability is defined as taking responsibility for one’s judgment and actions [19]. Therefore, it plays an essential role in nurses taking action in ethical decisions [18]. Moral sensitivity is the ability to become aware of patients’ vulnerability and recognize ethical conflicts. Thus, it is considered the first step in ethical decision-making [15, 16, 18, 20]. Furthermore, moral reasoning includes elucidating complex situations, finding the best solutions, and making decisions; moral agency is defined as recognition, reflection, and, ultimately, taking action on ones’ responsibilities [18]. Therefore, moral reasoning and moral agency contribute to converting the decision-making process from thought to practice. Meanwhile, moral practice is the ethical behavior that is the product of nurses’ ethical decision-making process [18]. When nurses fully complete this process, patients may experience a better death by making the optimal EOL decision.

Previous studies have examined the correlation between moral sensitivity and the moral components of ethical decision-making in a group of physicians or nurses. For example, Kim et al. [21] studied the relationship between nurses’ moral sensitivity and the implementation of the code of ethics. Similarly, Park et al. [22] identified that ethical education and nursing students’ moral sensitivity and reasoning were associated. However, most studies have not examined the whole ethical decision-making process; their focus was either on the thinking process (moral sensitivity, moral reasoning) or the behavioral process (moral practice). Moreover, despite the interest in EOL ethical issues, little research has been conducted on nurses’ ethical decision-making in the context of EOL care.

We aimed to identify nurses’ ethical decision-making process based on their moral sensitivity when caring for EOL patients. Specifically, this study’s research aims were (1) to identify the association between moral sensitivity and the ethical decision-making abilities of nurses who care for EOL patients; (2) to identify the factors that affect the ethical decision-making abilities of nurses in EOL circumstances.

## Methods

### Study design and participants

This study was a cross-sectional, descriptive survey and included 171 nurses selected by convenience sampling at a university hospital. The inclusion criteria were as follows: a nurse (1) with more than one year of experience, and (2) who currently works at a ward in the cancer center or intensive care unit (ICU), providing EOL care for patients.

### Measurements

We used the Korean version of the Moral Sensitivity Questionnaire (K-MSQ) to measure nurses’ moral sensitivity. It was initially developed by Lützén [20], and translated into Korean by Han [15]. The K-MSQ was reconstructed from the previous 30 questions to 27 questions, excluding three questions relevant to mental care. This instrument consists of five sub-dimensions: patient-oriented care, professional responsibility, conflict, meaning, and benevolence. It is measured on a seven-point scale; the higher the score, the higher the nurses’ moral sensitivity. At the time of development, the reliability of the K-MSQ using Cronbach’s alpha was 0.76. In this study, it was 0.83.

Nurses’ ethical decision-making ability was measured using the Nurses’ Ethical Decision-Making around End of Life Care Scale (NEDM-EOLCS) [18]. This instrument has 55 items on a six-point scale and consists of three sub-dimensions: perceived professional accountability, moral reasoning/moral agency, and moral practice. A higher score indicates a higher level of ethical decision-making ability. The internal consistency reliability of the NEDM-EOLCS using Cronbach’s coefficient alpha was 0.95 at the time of development. In the current study, the internal consistency was 0.96.

After questions on moral sensitivity and ethical decision-making, we asked general characteristics, such as age, gender, marital status, religion, and education level. Clinical experiences, department, and experiences of ethics education were also collected. Participants answered whether they experienced ethical conflicts within a week, and when they did, who can help them. Lastly, the participants scored their perceived work satisfaction and ethical conflict when they work on a 10-scale.

### Data collection and ethical considerations

We conducted this study after ethical approval was obtained from the Institutional Review Board of Yonsei University Health System (reference no. Y-2019-0111). In the internal board for nurses, the research purpose and method, risks and benefits of participation, confidentiality, and freedom to withdraw were explained before obtaining informed consent. After then, we got written consent from all voluntary participants. For the data collection, the authors followed guidelines by approved IRB protocol. A total of 196 questionnaires were distributed to ICUs and wards (111 and 85, respectively). The self-reported questionnaire was stored in a sealed envelope after being filled out, then collected by a researcher. This study only included the surveys of nurses who gave written informed consent and agreed to voluntary participation. All questionnaires we distributed were returned. However, twenty-four questionnaires were incomplete, and one respondent did not fulfill the inclusion criteria, which is more than one year of clinical experience. After removing them, 171 questionnaires were included in the final analysis.

### Data analysis

The collected data were analyzed by statistical software, R version 3.5.3 [23]. The participants’ general characteristics, moral sensitivity, and ethical decision-making were analyzed descriptively. The differences in moral sensitivity and ethical decision-making related to general characteristics were analyzed using the t-test and analysis of variance (ANOVA). The correlation coefficient between moral sensitivity and the sub-dimensions of ethical decision-making was calculated to identify their relation to each other. Multiple linear regression analysis was used to test the influence of moral sensitivity on nurses’ ethical decision-making. A two-sided *p*-value of less than .05 was considered statistically significant.

## Results

### General characteristics of the participants

The participants’ general characteristics are indicated in Table 1. The mean age was 33.3 years; 60.8% were single, and slightly more than half (52.4%) had a religion. The clinical characteristics were as follows: 52.1% worked at an ICU, while the remainder worked in the wards (oncology, palliative, or general unit in the cancer center). Most of the participants had experience in nursing ethics education (84.1%); in-hospital education was the most common type of ethics education (55.0%), followed by continuing education (34.5%). Approximately 30% of the participants experienced ethical dilemmas in the past week. When they faced an ethical dilemma, 67.8% of the sample stated that they asked for advice from their preceptors or charge nurses. The average job satisfaction and the perceived ethical conflict were 6.4 and 5.8 on a 10-point scale, respectively.

**Table 1:** General characteristics of participants (N = 171)

Variables	Categories	n (%)	Mean ± SD
Age (year)	20–29	79 (46.2)	33.3 ± 8.1 (years)
	30–39	52 (30.4)	
	≥ 40	40 (23.4)	
Gender	Male	9 (5.3)	
	Female	162 (94.7)	
Marital status	Single	104 (60.8)	
	Married	67 (39.2)	
Education (degree)	Bachelor’s degree or lower	151 (88.3)	
	Master’s degree or higher	20 (11.7)	
Religion	Yes	89 (52.4)	
	No	81 (47.6)	
Current clinical working area	Oncology/general/hospice Unit	81 (47.9)	
	Intensive care unit	88 (52.1)	
Total clinical experience	1–3 years	27 (15.8)	9.9 ± 8.0
	3–5 years	33 (19.3)	(years)
	5–10 years	52 (30.4)	
	> 10 years	59 (34.5)	
Current department experience	1–3 years	47 (29.4)	5.7 ± 5.4 (years)
	3–5 years	40 (25.0)	
	5–10 years	53 (33.1)	
	> 10yrs	20 (12.5)	
Experience in nursing ethics education	None	27 (15.9)	
	1–10 h	105(61.8)	
	> 10 h	38 (22.4)	
Type of nursing ethics education*	Continuing nursing education	59(34.5)	
	In-hospital education	94(55.0)	
	Academy/curriculum/others	18(10.5)	
	None	27(15.8)	
Experience of an ethical dilemma (within a week)	Yes	48 (29.6)	
	No	114 (70.4)	
Ethical decision support*	Colleagues	64(37.4)	
	Preceptor/charge nurse	116(67.8)	
	Nurse manager	36(21.1)	
	Others	3(1.8)	
Job satisfaction (0–10 point)	1–3	8 (4.7)	6.4 ± 1.6
	4–6	75 (43.9)	
	7–10	88 (51.5)	
Ethical conflict (0–10 point)	1–3	18 (10.5)	5.8 ± 1.7
	4–6	90 (52.6)	
	7–10	63 (36.8)	

*Multiple responses

### Level of moral sensitivity and ethical decision-making ability

The average mean of moral sensitivity was 4.8 ± 0.5 on a 7-point scale. In terms of ethical decision-making ability on a 6-point scale, the participants scored the highest in perceived professional accountability (5.2 ± 0.5) and the lowest in moral reasoning/moral agency (3.9 ± 0.6). The mean total ethical decision-making score was 4.6 ± 0.5 (Table 2).

**Table 2:** Moral sensitivity and ethical decision-making of participants

Variable (total number of items)	Mean ± SD	Average mean ± SD	Range
Moral sensitivity (27) (7-point scale)	125.3 ± 13.5	4.8 ± 0.5	91–160
Ethical decision-making (55) (6-point scale)	253.6 ± 27.3	4.6 ± 0.5	185–308
Perceived professional accountability (28)	144.5 ± 14.8	5.2 ± 0.5	97–167
Moral reasoning and moral agency (13)	47.0 ± 7.6	3.9 ± 0.6	27–70
Moral practice (14)	62.1 ± 9.2	4.4 ± 0.7	39–83

### Moral sensitivity and ethical decision-making process

Table 3 shows the differences in moral sensitivity and ethical decision-making related to the participants’ general characteristics. The ethical decision-making score of participants over 40 years old was significantly higher than others (*p* = 0.008), especially in moral reasoning/moral agency and moral practice (*p* = 0.004, *p* = 0.025, respectively). The nurses who have more than 10 years of clinical experience had higher moral reasoning/moral agency score (*p* = 0.007). The nurses who worked at oncology, general, or palliative care units had better ethical decision-making than ICU nurses. If the participants’ job satisfaction was over 7 points, their ethical decision-making score was significantly higher than others. Regarding the ethical conflict score, however, the lower-scoring group (1–3 points) and the higher-scoring group (7–10 points) scored better in ethical decision-making ability than the moderate-scoring group (4–6 points). Moreover, the higher-scoring group’s moral sensitivity was significantly higher than the others.

**Table 3:** Differences of moral sensitivity and ethical decision-making by general characteristics of the participants

Variables	Categories (n)	Moral sensitivity		Ethical decision-making		Sub-dimensions of ethical decision-making					
						Professional accountability		Moral reasoning/moral agency		Moral practice	
		Mean ± SD	*p*	Mean ± SD	*p*	Mean ± SD	*p*	Mean ± SD	*p*	Mean ± SD	*p*
Age (year)	≤ 29 (79)^a^ 30–39 (52)^b^ ≥ 40(40)^c^	123.8 ± 13.5 125.8 ± 12.6 128.1 ± 14.5	0.246	249.3 ± 28.5 251.4 ± 26.2 265.1 ± 23	0.008 a = b < c	142.4 ± 16.3 144.0 ± 14.0 149.2 ± 11.9	0.057	45.6 ± 7.5 46.7 ± 7.0 50.4 ± 7.6	0.004 a < c	61.3 ± 9.6 60.7 ± 8.9 65.5 ± 8.2	0.025 b < c
Gender	Male (9) Female (162)	122.8 ± 7.9 125.5 ± 13.7	0.551	245.9 ± 24.1 254.0 ± 27.5	0.386	140.1 ± 11.2 144.7 ± 15.0	0.367	44.8 ± 5.5 47.2 ± 7.7	0.360	61.0 ± 11.1 62.1 ± 9.1	0.717
Marital status	Single (104) Married (67)	123.8 ± 12.0 127.9 ± 15.3	0.067	249.2 ± 28.3 260.5 ± 24.4	0.008	142.1 ± 15.6 148.1 ± 12.9	0.009	46.0 ± 7.4 48.7 ± 7.6	0.022	61.1 ± 9.4 63.7 ± 8.8	0.074
Education (degree)	BSN or lower (151) MSN or higher (20)	125.3 ± 13.5 126.1 ± 14.0	0.805	252.7 ± 26.6 260.7 ± 32.5	0.217	144.0 ± 14.9 148.2 ± 14.7	0.228	46.8 ± 7.1 48.9 ± 10.4	0.390	61.9 ± 9.1 63.5 ± 10.2	0.452
Religion	Yes (89) No (81)	126.3 ± 13.7 124.6 ± 13.3	0.430	255.1 ± 28.5 252.6 ± 25.6	0.537	145.5 ± 14.7 143.8 ± 14.5	0.437	47.1 ± 8.1 47.0 ± 7.0	0.890	62.5 ± 9.3 61.8 ± 9.1	0.637
Current clinical department	Oncology/general/hospice Unit (81) Intensive Care Unit (88)	127.5 ± 13.5 123.7 ± 13.4	0.067	260.7 ± 28.6 247.2 ± 24.8	0.001	148.0 ± 14.7 141.2 ± 14.4	0.003	48.3 ± 8.6 45.8 ± 6.3	0.034	64.3 ± 9.1 60.1 ± 8.9	0.003
Total clinical experience (yrs)	1–5 years (60)^a^ 5–10 years (52)^b^ > 10 years (59) ^c^	123.4 ± 13.0 126.0 ± 13.8 126.9 ± 13.8	0.347	249.5 ± 28.2 248.9 ± 28.0 261.9 ± 24.2	0.015 a = b < c	142.5 ± 16.0 142.1 ± 15.5 148.6 ± 12.2	0.030 a = b = c	46.0 ± 7.3 45.5 ± 7.0 49.5 ± 7.8	0.007 a = b < c	61.0 ± 9.4 61.4 ± 9.9 63.8 ± 8.3	0.223
Current department experience (yrs)	0–5 years (96) 5–10 years (53) > 10 years (20)	124.6 ± 13.0 126.0 ± 14.1 126.0 ± 14.1	0.790	251.9 ± 28.1 253.9 ± 28.8 260.8 ± 17.1	0.412	143.6 ± 15.0 144.8 ± 15.9 148.2 ± 10.4	0.438	46.7 ± 7.7 46.8 ± 7.9 49.2 ± 5.7	0.382	61.6 ± 9.7 62.2 ± 9.3 63.3 ± 6.6	0.725
Experience in nursing ethics education	None (27)^a^ 1–10 h (105)^b^ > 10 h (38)^c^	123.7 ± 11.2 124.1 ± 13.0 130.2 ± 15.5	0.041 b < c	237.6 ± 19.7 251.2 ± 27.6 270.5 ± 21.8	< 0.001 a < b < c	137.0 ± 12.2 143.8 ± 15.6 151.2 ± 11.4	< 0.001 a = b < c	43.0 ± 4.7 45.9 ± 7.2 52.6 ± 7.3	< 0.001 a = b < c	57.5 ± 7.2 61.5 ± 9.7 66.8 ± 7.0	< 0.001 a = b < c
Experience of ethical dilemma											
(within a week)	Yes (48) No (114)	130.7 ± 15.1 123.7 ± 12.3	0.002	256.4 ± 27.8 252.7 ± 27.3	0.436	144.2 ± 15.5 144.4 ± 14.9	0.933	48.3 ± 7.5 46.8 ± 7.6	0.231	63.9 ± 9.2 61.6 ± 8.9	0.131
Job satisfaction	1–3 (8)^a^ 4–6 (75)^b^ 7–10 (88)^c^	123.1 ± 5.9 123.1 ± 12.5 127.6 ± 14.5	0.088	240.1 ± 26.2 244.8 ± 27.9 262.4 ± 24.1	< 0.001 b < c	138.8 ± 15.3 139.9 ± 16.6 148.9 ± 11.7	< 0.001 b < c	41.6 ± 6.6 45.8 ± 6.8 48.6 ± 7.9	0.007 a < c	59.8 ± 9.1 59.1 ± 9.2 64.8 ± 8.5	< 0.001 b < c
Ethical conflict score	1–3 (18)^a^ 4–6 (90)^b^ 7–10 (63)^c^	121.1 ± 11.3 121.1 ± 11.6 132.8 ± 13.6	< 0.001 a = b < c	268.1 ± 20.4 245.1 ± 29.1 261.6 ± 22.1	< 0.001 b < a = c	154.2 ± 11.4 140.6 ± 16.1 147.2 ± 11.8	< 0.001 b < a = c	47.6 ± 7.1 45.4 ± 7.5 49.2 ± 7.3	0.008 b < c	66.2 ± 5.8 59.1 ± 9.5 65.2 ± 8.1	< 0.001 b < a = c

All sub-dimensions of ethical decision-making and moral sensitivity showed significant correlations with each other. The moral practice was the highest correlated sub-dimension to moral sensitivity (r = 0.49, *p* < 0.001), followed by perceived professional accountability and moral reasoning/moral agency (r = 0.43, *p* < 0.001; r = 0.37, *p* < 0.001, respectively).

### Factors affecting the ethical decision-making process

The regression model used to identify factors related to the ethical decision-making process is described in Table 4. The variables entered into the regression model were moral sensitivity, age, education level, clinical department, experience of ethical education, job satisfaction, and ethical conflict. We chose the variables with a *p*-value less than 0.05 to enter the multiple linear regression model. As marital status and clinical experience were highly associated with age, we did not include these two variables in the final model. Education level was included based on the literature [24]. The multiple linear regression analysis revealed that moral sensitivity, clinical department, the experience of ethics education, job satisfaction, and ethical conflict score explained a significant amount of variance in ethical decision-making (R^2^ = 0.459, *p* < 0.001). To be specific, ethics education for more than 10 h had a significant favorable influence on ethical decision-making (β = 20.45, *p* < 0.001). Moreover, job satisfaction and moral sensitivity also positively affected ethical decision-making (β = 5.27, *p* < 0.001; β = 0.85, *p* < 0.001, respectively). In contrast, a higher ethical conflict score harmed ethical decision-making (β =  − 2.260, *p* < 0.031).

**Table 4:** Influential factors of ethical decision-making

Variables	β	Standard error	*p*-value	95% confidence interval
Moral sensitivity	0.852	0.134	< 0.001	0.587, 1.117
Age	0.166	0.220	0.450	− 0.268, 0.600
*Education level (ref. BSN or lower)*				
MSN or higher	− 4.456	5.447	0.415	− 15.214, 6.302
*Department (ref. wards)*				
ICU	− 7.018	3.291	0.035	− 13.517, − 0.518
*Ethics education (ref. none)*				
1–10 h	10.462	4.551	0.023	1.472, 19.452
> 10 h	20.450	5.689	< 0.001	9.214, 31.686
Job satisfaction	5.273	1.119	< 0.001	3.064, 7.483
Ethical conflict score	− 2.260	1.040	0.031	− 4.315, − 0.205

(Multiple R-squared: 0.4585, Adjusted R-squared: 0.4312)

BSN: Bachelor of Science in Nursing, MSN: Master of Science in Nursing, ICU: Intensive Care Unit

## Discussion

In this study, we revealed a gap between the thinking and behavior of the ethical decision-making process of nurses. Although ethical decision-making is a continuous sequential process that is not divided dichotomously, it seems that there are some impediments until moral actions occur. Even if the survey cannot fully reflect real-life behavior in ethical decision-making, it can reflect nurses’ intention to act. Therefore, how to reduce this discordance must be discussed to improve nurses’ ethical decision-making during EOL care. Further, how moral sensitivity and other factors affect the ethical decision-making process should be considered.

This study showed that participants scored highest in professional accountability (5.2 ± 0.5) and lowest in moral reasoning/moral agency (3.9 ± 0.6). For this reason, the ethical decision-making process was less likely to lead to the next step, a moral practice (4.4 ± 0.7). If the gap between professional accountability and moral reasoning/moral agency is widened further, there would be a negative influence on moral practice. Moreover, when nurses who have high moral sensitivity and professional accountability fail to make appropriate ethical decisions, they may experience frustration and exhaustion [25], which would negatively affect their quality of care [17]. Thus, decreasing the gap between the thinking and behavioral processes of nurses’ ethical decision-making is of the utmost importance. This means that an individual and organizational effort to improve nurses’ moral reasoning/moral agency ability is required. For example, clinical ethics support services such as clinical ethics consultations and clinical ethics committees could help medical staff with moral reasoning difficulties improve their ethical decision-making [26].

Moral sensitivity is a personal attribute that plays an essential role in nurses’ ethical decision-making. According to this study, moral sensitivity positively correlated with ethical decision-making. Previous research conducted by Lützén et al. explained that clinical experience could develop moral sensitivity [20]. This research also argued that various aspects of moral sensitivity, such as respect for the patient’s autonomy, improved with age, regardless of where they worked [20]. In the present study, however, there were no significant differences in moral sensitivity among the different groups according to age or clinical experience. The variables significantly related to moral sensitivity were the experience of ethical dilemma, nursing ethics education, and perceived ethical conflict.

This finding might be because people with high moral sensitivity could identify ethical conflicts or ethical dilemmas well. On the other hand, nurses could become morally sensitive by experiencing various ethical conflicts. In this context, moral case deliberation (MCD), a systematic approach in real clinical cases to support healthcare personnel to deal with ethical conflict [26], could improve nurses’ moral sensitivity. Exposing them to various clinical cases could broaden their perspectives on ethical dilemmas.

The regression model depicted in Table 4 shows that ICU nurses had a lower score than those of oncology/palliative/general ward nurses, which implies highly different environments in ICUs and wards. It is generally believed that ICUs are not an appropriate place to provide EOL care because it was not designed for such scenarios [2, 27]. ICUs tend to focus on providing intensive care for critically ill patients to reduce mortality [27]. Moreover, they do not guarantee a comfortable environment, private rooms, and sufficient time with families for practical reasons. Besides, most ICU patients cannot make their own EOL decisions and tend to depend on family members (or surrogates) to do so [28]. ICU physicians and nurses are less likely to have opportunities to interact with patients or their families than physicians and nurses in the wards, so there is a greater risk of discordance between healthcare providers’ and patients’ values and goals [29]. Nonetheless, ICU nurses have frequently faced terminally ill patients and EOL decision-making situations. Therefore, specific ethics training opportunities for ICU nurses are needed to improve their ethical decision-making. There have been many efforts to improve palliative care in ICUs. For example, the Improving Palliative Care in the ICU (IPAL-ICU) Project provides useful information for palliative care in the ICU, such as practical tools and links to professional education curricula [30]. This is a worthy attempt to solve the ethical issues in the ICU and increase ICU nurses’ ethical competency. As such, ongoing efforts are necessary to reduce ethical problems in the ICU.

Lack of knowledge or education is the most common reason for ethical dilemmas [29]. As knowledge is an essential component to identify ethical problems and perform ethical decision-making [22], ethics in the clinical health care setting is performed based on knowledge. Our finding showed that the participants who had taken ethics educations had higher ethical decision-making scores than those who never took ethics education. This result supports that experience of ethics education is an influential factor in the ethical decision-making process. According to a study that addressed the need for clinical nurses’ ethics education in Korea, “patient rights, autonomy, and advance directives” was the most needed topic in ethics education [31]. Therefore, as the refined curriculum considering their needs would improve their ethical decision-making, the nurses’ demand for ethics education must be continuously identified. Admittedly, education alone does not change practice. Nevertheless, education plays an important role in improving moral sensitivity that nurses can recognize ethical issues [22]. After that, organizational efforts are required for a sequential process from moral reasoning to action, and they can contribute to making an ethical climate in consequence.

Job satisfaction also had a significant positive impact on ethical decision-making. As one of the factors related to nurses’ job satisfaction, the ethical climate contributes toward making nurses feel more satisfied with their jobs [25, 32]. In cases where there was a congruence between ethical codes and organizational policies, nurses felt that they were working in an ideal moral environment. Thus, creating an ethical working environment could also be considered essential to improving nurses’ ethical decision-making.

There are some limitations to this study. First, convenience sample collection in a hospital makes this study challenging to apply to all population groups. Second, moral sensitivity and perceived accountability, a sub-dimension of nurses’ ethical decision-making, have a partial overlap. Therefore, this may have affected the outcome of their correlation and the regression analysis. Moreover, as all the findings are based on the self-perceived questionnaire, there is a possibility that the findings could not entirely reflect the actual phenomenon. Therefore, interpreting these results, subjectivity should be considered. We suggest further study to observe nurses’ real-life moral practice or investigate patients and their families’ responses who are given nurses’ EOL care. Additionally, confounding factors affecting nurses’ ethical decision-making process and the generalization of its relationship with moral sensitivity should be appropriately controlled in further studies.

## Conclusion

In this study, nurses’ general ethical decision-making was more than at a moderate level. The results imply that moral sensitivity could positively affect nurses’ ethical decision-making during EOL care. Other significant factors that affect ethical decision-making were the clinical department, the experience of ethics education, job satisfaction, and ethical conflict. Nurses’ ethical decision-making in EOL care is essential to ensure that patients pass away peacefully in hospitals. If nurses’ ethical decision-making process were improved, they could make better EOL decisions during ethical dilemmas and provide patients with a better quality of death.

## Data Availability

The data supporting the findings are available from the corresponding author on request by the journal.

## Abbreviations

EOL: End of life
LST: Life-sustaining treatment
ICU: Intensive care unit
K-MSQ: The Korean version of the moral sensitivity questionnaire
NEDM-EOLCS: Nurses’ ethical decision-making around end of life care scale
